# Case Report: Making room for radicality: balancing liver reserve and oncologic clearance in hilar cholangiocarcinoma through portal vein embolisation

**DOI:** 10.3389/fsurg.2026.1830529

**Published:** 2026-07-08

**Authors:** Elvin Jia Hong Lee, Zhun Shen Tan, Azlanudin Azman, Isa Azzaki Zainal, Muhammad Zahid Abdul Muien, Ian Chik

**Affiliations:** 1 Faculty of Medicine, National University of Malaysia, Kuala Lumpur, Malaysia; 2 Hepatobiliary & Pancreatic Surgery Unit, Department of Surgery, Faculty of Medicine, National University of Malaysia, Kuala Lumpur, Malaysia; 3 Department of Radiology, Faculty of Medicine, Hospital Canselor Tuanku Muhriz, Universiti Kebangsaan Malaysia Medical Centre, Kuala Lumpur, Malaysia; 4 Radiology Department, Faculty of Medicine and Health Sciences, Universiti Malaysia Sabah, Kota Kinabalu, Sabah, Malaysia

**Keywords:** case report, extended hepatectomy, future liver remnant, hilar cholangiocarcinoma, klatskin tumor, portal vein embolization, posthepatectomy liver failure, resection margin

## Abstract

Hilar cholangiocarcinoma frequently necessitates extended hepatectomy to achieve oncological clearance, yet the risk of post-hepatectomy liver failure (PHLF) remains a critical concern when the future liver remnant (FLR) is inadequate. Over the last decade, strategies to induce FLR hypertrophy, including Portal Vein Embolization (PVE), Liver Venous Deprivation (LVD), and Associating Liver Partition and Portal Vein Ligation for Staged Hepatectomy (ALPPS) have redefined the boundaries of resectability in patients with limited FLR. This report discusses a successful extended right hepatectomy enabled by PVE and explores the technical and physiological considerations of modern liver hypertrophy techniques for advanced hilar cholangiocarcinoma, highlighting three key aspects of contemporary hepatobiliary surgery: the physiological role of FLR optimization, the evolving hierarchy of liver hypertrophy strategies, and the intraoperative balance between oncologic radicality and preservation of functional liver reserve. We present a case of a 61-year-old male patient with minimal symptoms who was incidentally diagnosed with Bismuth-Corlette type IIIA hilar cholangiocarcinoma following detection of elevated serum carbohydrate antigen 19-9 (CA19-9) and cholestatic liver dysfunction. Multidisciplinary evaluation identified marginal FLR of 34%, prompting right PVE. Post-PVE volumetric assessment demonstrated robust hypertrophy with FLR increasing to 60.5%. The patient subsequently underwent open extended right hepatectomy with caudate lobectomy, complete bile duct excision, regional lymphadenectomy and Roux-en-Y hepaticojejunostomy reconstruction. The hypertrophied FLR enabled safe completion of extended resection without PHLF. This underscores the importance of preoperative PVE in converting a marginal FLR into an adequate physiological reserve, thereby enabling safe completion of extended hepatectomy for hilar cholangiocarcinoma without PHLF.

## 1 Introduction

Hilar cholangiocarcinoma, also known as perihilar cholangiocarcinoma (pCCA) or Klatskin tumor, arises at the biliary confluence and accounts for approximately 50%–60% of all cholangiocarcinomas. These tumors are characterized by insidious onset, late clinical presentation, and a propensity for longitudinal submucosal spread along the biliary epithelium, frequently extending beyond radiographic boundaries. Consequently, curative treatment often requires extended hepatectomy with caudate lobectomy and bile duct resection to achieve negative margins (1). Despite advances in surgical technique and perioperative care, post-hepatectomy liver failure (PHLF) remains the principal cause of morbidity and mortality following major liver resection. The risk is particularly significant in patients with hilar cholangiocarcinoma, where underlying cholestasis, biliary infection, and chronic liver disease may impair hepatic regenerative capacity. As a result, the adequacy of the FLR has emerged as the most critical physiological determinant of resectability (2). PVE has become an established strategy to induce hypertrophy of the remnant liver prior to major hepatectomy. By redirecting portal blood flow toward the intended remnant, PVE stimulates hepatocellular proliferation and functional adaptation, thereby expanding the limits of safe resection. In recent years, additional hypertrophy techniques, including combined portal and hepatic vein embolization (PVE/HVE) and associating liver partition and portal vein ligation for staged hepatectomy (ALPPS), have further extended the boundaries of surgical management in patients with marginal FLR (3–5). This case report describes a patient with Bismuth-Corlette type IIIA hilar cholangiocarcinoma in whom preoperative PVE enabled safe extended right hepatectomy. We also present our centre's initial experience with hepatectomy following preoperative PVE, a technique that is still infrequently performed not just in our institution, but also in the wider national hepatobiliary surgical landscape.

## 2 Case presentation

A timeline summarizing the key clinical events is provided in Table 1.

**Table 1:** Timeline of clinical events.

Time from Presentation	Clinical Event	Key Laboratory/ Imaging Data
Day 0 (Baseline)	Initial presentation with abnormal routine health screening	CA19-9: 43 U/mL, Bilirubin: 36.7 μmol/L, ALP: 966 U/L, GGT: 3354 U/L, AST 89 U/L, and ALT 170 U/L. Synthetic function was preserved, with albumin 39 g/L, INR 1.05, and platelet count 235 × 10⁹/L.
Week 1	CECT and MRCP diagnosis: Bismuth IIIA hilar cholangiocarcinoma	FLR: 34% (493 mL/1450 mL TLV)
Week 2	Multidisciplinary team: Decision for PVE	Albumin: 39 g/L, INR: 1.05, Platelet: 235 × 10⁹/L
Week 3	Right PVE performed	Procedure completed without complications
Week 6	Post-PVE CT volumetry and surgical planning	FLR: 60.5% (878 mL/1450 mL), 78% hypertrophy, Bilirubin: 32.1 μmol/L
Week 7 (Operation)	Extended right hepatectomy with caudate lobectomy and biliary reconstruction	Operative time: 7 h 15 min, Estimated Blood Lost: 700 mL, R0 resection achieved
POD 3-5		Peak bilirubin: 48 μmol/L (POD 4), Drain output: 500–800 mL/day
POD 9	Drain removal	Bilirubin improving: 38 μmol/L
POD 10	Hospital discharge	Stable liver function, good oral intake
Week 13	Initiation of adjuvant capecitabine chemotherapy	Cycle 1 of planned 8 cycles commenced
Month 6	Latest follow-up: completion of 6th out of 8 chemotherapy cycles	CT scan: No recurrence, liver remnant: continued hypertrophy, Bilirubin: 15 μmol/L, ECOG PS: 0
Ongoing	Adjuvant chemotherapy and surveillance	Planned completion of 8 cycles with regular imaging follow-up

ALP: alkaline phosphatase, GGT, gamma-glutamyl transferase; AST, aspartate aminotransferase; ALT, alanine aminotransferase; CECT, contrast-enhanced computed tomography; MRCP, magnetic resonance cholangiopancreatography; TLV, total liver volume; POD, post-operation day.

### 2.1 Patient information and clinical findings

A 61-year-old male (ECOG performance status 0) was referred to us following routine health screening that demonstrated mildly elevated serum carbohydrate antigen 19-9 (CA19-9, 43 U/mL) and a cholestatic pattern of liver enzyme derangement. He did not initially seek medical attention for symptomatic complaints. Upon further history taking during evaluation, he reported a two-week history of tea-colored urine but denied overt jaundice, abdominal pain, fever, weight loss, anorexia, or constitutional symptoms. His medical history was unremarkable. Physical examination was normal, without stigmata of chronic liver disease or palpable hepatomegaly.

Laboratory evaluation revealed total bilirubin 36.7 µmol/L, ALP 966 U/L, GGT 3354 U/L, AST 89 U/L, and ALT 170 U/L. Synthetic function was preserved, with albumin 39 g/L, INR 1.05, and platelet count 235 × 10⁹/L. Hepatitis B serology was consistent with chronic infection. Carcinoembryonic antigen (CEA) and alpha-fetoprotein (AFP) were within normal limits.

### 2.2 Diagnostic assessment

CECT and MRCP demonstrated a 2.5 cm hilar mass involving the right hepatic duct and biliary confluence, with preservation of the left hepatic duct origin. No vascular encasement, regional lymphadenopathy, or distant metastasis was identified. The imaging findings were consistent with Bismuth-Corlette type IIIA hilar cholangiocarcinoma (Figure 1).

**Figure 1:**
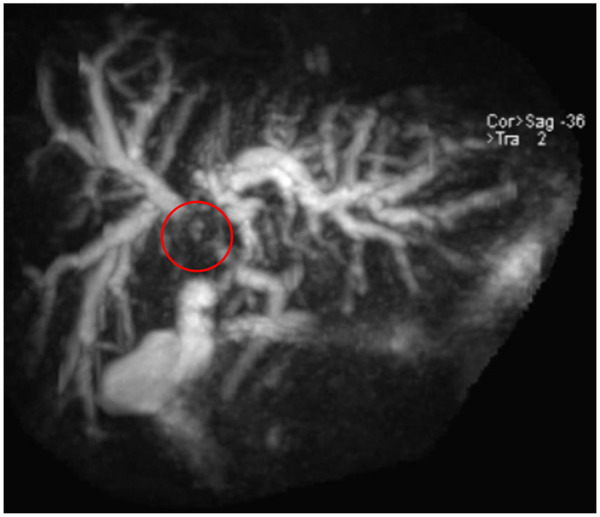
MRCP with a red circle highlighting a hilar tumor at the biliary confluence causing compression of the proximal bile ducts, resulting in focal signal loss and upstream intrahepatic biliary dilatation.

In our case, preoperative biliary drainage was deferred as the patient's total bilirubin remained stable at 36.7 μmol/L. This is supported by a multicentre European study which showed most hepatobiliary centers reserve preoperative biliary drainage for patients with total bilirubin exceeding 50–100 μmol/L, particularly when extended hepatectomy or prolonged waiting period for PVE is anticipated. Below this threshold, especially when synthetic liver function is preserved, biliary drainage may not provide additional benefit but carries procedure-related risks, therefore no clear evidence of improved surgical outcomes (6).

Another evidence highlighted that ERCP in hilar cholangiocarcinoma carries risks of tumor seeding along the instrumentation tract and procedure-related complications including pancreatitis and perforation (7).

Given the stable bilirubin, preserved synthetic function, and plan for prompt PVE followed by resection, a multidisciplinary decision was made to proceed without biliary drainage and ERCP. Diagnosis was established based on imaging and clinical context following multidisciplinary consensus involving hepatobiliary surgeons, radiologists, and oncologists.

#### 2.2.1 Volumetric analysis methodology

Preoperative volumetric assessment was performed using OsiriX MD imaging software (Pixmeo SARL, Geneva, Switzerland) on portal venous phase CECT images.

Total liver volume (TLV) was estimated using Vauthey’s standardized liver volume formula based on body surface area (BSA), rather than direct whole-liver segmentation.

#### 2.2.2 Vauthey formula (estimate TLV = 18.51 × body weight + 191.8)

Future liver remnant (FLR) volumetry was then performed by manual segmentation on sequential axial CT slices at 5-mm intervals. As right hepatectomy was planned, the middle hepatic vein (MHV) served as the anatomical transection landmark, and all functional liver parenchyma to the left of the MHV was included in the FLR calculation. Major vascular structures and intrahepatic bile ducts were excluded from volumetric measurements. (Figure 2).

**Figure 2:**
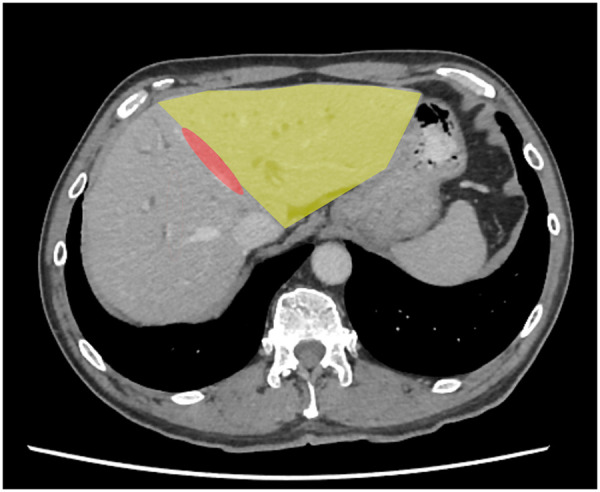
Pre-PVE CECT demonstrating baseline FLR volumetry. The MHV (red overlay) served as the anatomical transection landmark for the planned right hepatectomy. All functional liver parenchyma to the left of the MHV (yellow overlay) was included in the FLR calculation, corresponding to a baseline FLR of 34% according to standardized volumetric assessment.

Following right PVE, repeat CT volumetry was performed using the same standardized TLV derived from Vauthey's formula. Post-PVE FLR was recalculated using identical anatomical boundaries to assess hypertrophy of the remnant left liver.

#### 2.2.3 Regarding functional liver assessment

We acknowledge that functional volumetric assessment such as using indocyanine green (ICG) clearance testing was not performed in this patient. While functional assessment is valuable, particularly in cholestatic patients with underlying liver disease, it is not available at our institution at the time of this patient's management. Our preoperative assessment relied on volumetric analysis combined with clinical parameters including synthetic liver function (albumin, INR, platelet count), degree of hyperbilirubinemia, and absence of clinical signs of portal hypertension or cirrhosis. Post-PVE, the robust volumetric hypertrophic response (78% increase) was taken as an indirect indicator of preserved hepatic regenerative capacity and functional reserve.

### 2.3 Therapeutic intervention

Right PVE was performed on 12 November 2025, via an ipsilateral transhepatic approach using gelatin sponge particles and coils, including embolisation of the segment IV branch. The procedure was uneventful. Three-week follow-up computed tomography demonstrated marked hypertrophy of the left lateral section to 878 mL, corresponding to an FLR of 60.5%, representing a 78% relative increase (Figure 3). Liver function remained stable, and the patient was cleared for surgery.

**Figure 3:**
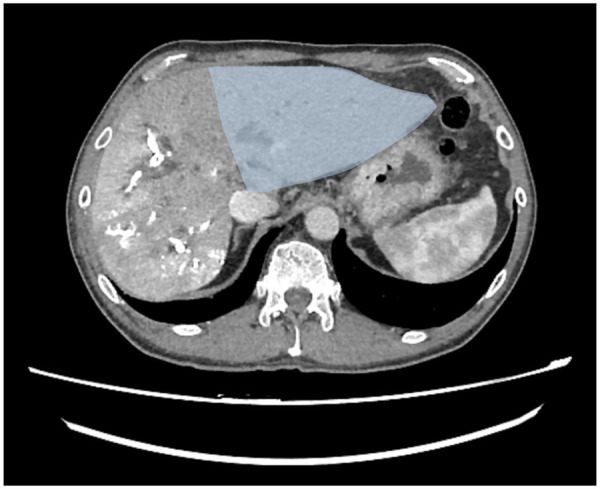
CECT performed three weeks after right PVE. Multiple hyperdense embolic materials seen within the right portal vein branches. Blue overlay: hypertrophy of the left liver remnant.

On 8 December 2025, the patient underwent open extended right hepatectomy with *en bloc* caudate lobectomy, extrahepatic bile duct excision, regional lymphadenectomy, and Roux-en-Y hepaticojejunostomy reconstruction (Figure 4). Intraoperative ultrasound excluded occult intrahepatic lesions. For frozen section analysis, The first sample was obtained from the proximal common bile duct at the hilar confluence, demonstrating malignant cells, indicating persistent proximal tumor extension. Additional proximal ductal trimming was subsequently undertaken. Repeat frozen section from this second proximal margin showed atypical cells, prompting further extension. Trimming of the left intrahepatic duct (IHD) was then done further till maximum extent (second order of left IHD) for histopathological examination (HPE), which confirmed negative margin. (R0 resection).

**Figure 4:**
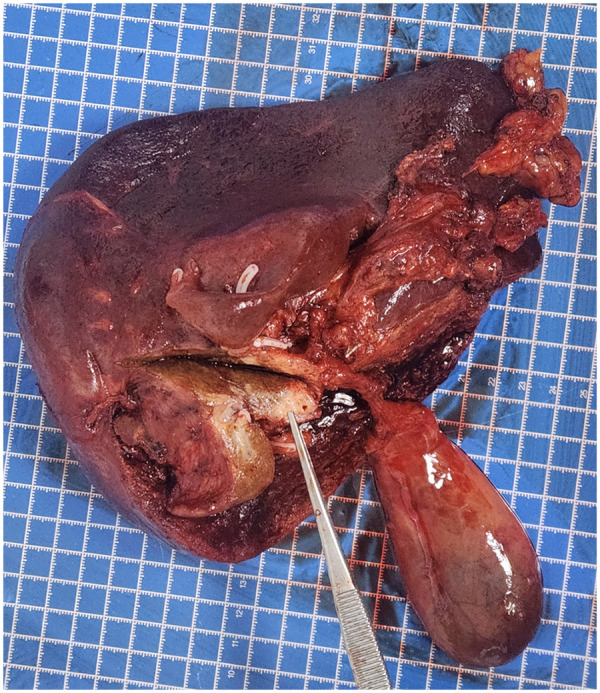
Resected liver specimen with gallbladder and exposed hepatic hilum. Forceps pointing a mass at the biliary confluence extending into the right hepatic duct.

Parenchymal transection was performed using the crush-clamp technique with intermittent Pringle maneuver (15 min clamping, 5 min release, three cycles). There were three bile duct openings, but two were combined as a single hepaticojejunostomy, constructed to the segment II and III ducts,with modified silicone tube used as a stent. Operative time was 7 h 15 min, estimated blood loss was 700 mL, and no transfusion was required.

### 2.4 Follow-up and outcomes

Early postoperative course: Recovery was hemodynamically stable without bile leak, surgical site infection, or hepatic decompensation. The patient did not meet ISGLS biochemical criteria for PHLF. Drains were removed on POD 9, and the patient was discharged in stable condition on POD 10. Final histopathology demonstrated a 2.8 × 2.5 cm well-differentiated hilar cholangiocarcinoma involving the hilar confluence with right ductal extension. All margins were negative (R0 resection), with no nodal metastasis (0/12). Final staging was pT1aN0M0 (AJCC 8th edition).

#### 2.4.1 Adjuvant therapy and follow-up

Following multidisciplinary discussion, adjuvant capecitabine was initiated for 8 planned cycles. At latest follow-up, the patient had completed 6 cycles with good tolerance and preserved functional status (ECOG 0). At 6 months, CECT showed no local recurrence or distant metastasis and CT volumetry demonstrated continued hypertrophy of the liver remnant. Ongoing surveillance includes clinical review, tumor markers (CA19-9, CEA), and cross-sectional imaging following completion of adjuvant therapy.

## 3 Discussion

### 3.1 The physiological role of FLR optimization via PVE

In hilar cholangiocarcinoma, the extent of hepatic resection is determined primarily by biliary ductal involvement rather than tumor bulk, meaning the surgical field frequently exceeds the radiographically visible lesion. Achieving oncologic clearance therefore often requires extended hepatectomy with caudate lobectomy, particularly in Bismuth type III-IV disease. In this setting, the adequacy of the FLR becomes the principal physiological determinant of resectability. Strategies that induce compensatory hypertrophy of the FLR, most notably PVE, have therefore become fundamental tools in modern hepatobiliary surgery, allowing surgeons to balance oncologic radicality with preservation of hepatic reserve. PVE works by inducing compensatory hypertrophy by redirecting portal flow toward the remnant liver, stimulating hepatocellular proliferation through increased portal shear stress and hepatotrophic signaling (1).

Current consensus suggests FLR thresholds of approximately 20% in normal liver, ≥ 30% in chemotherapy-exposed liver, and ≥ 40% in cholestatic or diseased liver (1, 3). These are particularly relevant in hilar cholangiocarcinoma, where cholestasis and chronic liver disease impair reserve. In this patient, the baseline FLR of 34% was marginal given cholestatic dysfunction and chronic hepatitis B. PVE increased FLR from 34% to 60.5% within three weeks, a robust 78% hypertrophic response that converted a borderline resection into a physiologically safe procedure while minimizing risk of tumor progression during the waiting interval (2).

Beyond volumetric augmentation, PVE provides a dynamic physiologic stress test. The rate of hypertrophy reflects regenerative capacity, with kinetic growth rate (KGR) serving as a predictor of postoperative outcomes. Inadequate hypertrophy signals increased risk of liver failure, whereas robust growth indicates preserved regenerative potential (3, 4). The interval following PVE should be viewed as a structured preoperative phase for reassessment of tumor biology, hepatic function, and surgical anatomy, allowing optimization of nutritional status and confirmation of regenerative response prior to definitive surgery (2).

### 3.2 The evolving hierarchy of liver hypertrophy strategies: PVE/HVE and ALPPS

While conventional PVE achieves sufficient hypertrophy in most patients, inadequate or delayed FLR growth occurs in some cases. Combined portal and hepatic vein embolization (PVE/HVE), also termed liver venous deprivation (LVD), addresses this by simultaneously occluding portal inflow and hepatic venous outflow. Multicenter studies demonstrate that PVE/HVE produces greater and more rapid hypertrophy with higher KGR and shorter intervals to surgery (5, 8). However, the technique is more complex and may carry higher risk of biliary complications, requiring careful patient selection. Another strategy, Associating liver partition and portal vein ligation for staged hepatectomy (ALPPS) induces extremely rapid hypertrophy within 1–2 weeks but remains controversial in hilar cholangiocarcinoma. Systematic analyses show high morbidity and mortality when ALPPS is applied to hilar tumors, likely reflecting underlying cholestasis, infection risk, and reconstruction complexity (9, 10). Most centers therefore advocate a stepwise strategy: conventional PVE as first-line, escalation to PVE/HVE for cases requiring more rapid or substantial hypertrophy, and ALPPS reserved for highly selected salvage situations. This graduated approach allows surgeons to tailor hypertrophy strategies according to patient physiology while minimizing procedural risk. In our case, favorable anatomy and absence of factors necessitating aggressive hypertrophy supported selection of conventional PVE.

### 3.3 Surgical strategy and the margin dilemma

Margin status remains one of the strongest oncologic determinants of long-term survival in hilar cholangiocarcinoma, yet the pursuit of negative margins must be balanced against the physiological limits of hepatic resection. Hilar cholangiocarcinoma spreads longitudinally along the biliary epithelium and perineural planes, often extending beyond radiographic boundaries. As a result, intraoperative frozen section analysis of the proximal bile duct margin is essential and frequently necessitates sequential proximal trimming to achieve ductal clearance (11). However, each additional ductal resection increases operative complexity and the demands of biliary reconstruction. This margin dilemma therefore lies in determining how aggressively to pursue ductal clearance without compromising hepatic reserve or biliary drainage of the remnant liver. For the present case, frozen section assessment initially demonstrated malignant cells at the proximal bile duct margin, necessitating stepwise advancement which were finally cleared of malignant cells. Ultimately, two hepaticojejunostomies were constructed to maintain drainage of the remnant segments. Thus, achieving negative margins required careful proximal trimming while preserving sufficient ductal structures for reconstruction. Importantly, aggressive additional proximal ductal resection may not necessarily confer oncologic benefit. Yoshihiro Shingu et al. reported that once maximal or near-maximal ductal transection had been achieved, further re-resection of the proximal bile duct beyond 5 mm did not improve survival and was associated with increased postoperative complications, suggesting that excessive pursuit of ductal clearance should be approached cautiously (12). Equally important is the distinction between ductal and radial margins. While ductal margins can be extended through further trimming, radial margins at the hepatic hilum are constrained by adjacent vascular structures such as the portal vein and hepatic artery. For this reason, caudate lobectomy and complete extrahepatic bile duct excision remain integral components of oncologic resection, enabling circumferential clearance of the biliary confluence and surrounding perihilar tissue planes. On this basis, the ability to pursue this margin strategy safely was fundamentally enabled by preoperative FLR optimization. By expanding the physiologic reserve of the remnant liver, PVE widened the operative window in which oncologic radicality could be achieved without exceeding the limits of hepatic tolerance.

### 3.4 Limitations

This case report has inherent limitations as a single-patient observation. While it demonstrates the successful application of PVE to enable extended hepatectomy, the outcome cannot be generalized to all patients with hilar cholangiocarcinoma. Clinical outcomes are influenced by multiple factors, including patient selection, tumor biology, baseline liver function, surgical expertise, and the quality of perioperative care. Future directions in the management of hilar cholangiocarcinoma include further refinement of patient selection criteria for PVE vs. alternative hypertrophy strategies such as PVE/HVE and ALPPS. In addition, the development of validated predictive models for PHLF incorporating both preoperative and intraoperative variables may improve perioperative risk stratification. Ongoing multicenter collaborative studies will be essential to generate robust evidence and guide optimal management in this relatively rare and complex disease.

## 4 Conclusion

This case illustrates the central role of future liver remnant optimization in enabling curative-intent resection for hilar cholangiocarcinoma. PVE remains the cornerstone strategy for patients with marginal FLR, providing both volumetric hypertrophy and a physiologic assessment of hepatic regenerative capacity prior to major hepatectomy. The management of patients with insufficient FLR should be approached using a stepwise framework of hypertrophy strategies. Conventional PVE remains the first-line due to its safety profile and predictable regenerative response, while escalation techniques such as PVE/HVE or ALPPS may be considered in selected patients requiring more rapid or substantial hypertrophy. Finally, this case underscores the importance of intraoperative margin assessment and tailored biliary reconstruction in hilar cholangiocarcinoma surgery. Achieving R0 resection often requires sequential proximal ductal advancement guided by frozen section, while preserving adequate biliary drainage of the remnant liver. Preoperative FLR optimization plays a critical enabling role in this process by expanding the physiological margin within which oncologic radicality can be safely pursued. Taken together, these principles highlight the evolving integration of physiologic optimization, staged hypertrophy strategies, and meticulous surgical technique in the contemporary management of hilar cholangiocarcinoma.

## 5 Patient perspective

The patient noted that the preoperative PVE procedure was well-tolerated. He appreciated the thorough preoperative explanation and felt confident in the treatment plan. At his six-month follow-up visit, he reported excellent functional recovery with return to all normal activities including full-time work. He experienced no significant limitations in daily life and expressed high satisfaction with both the surgical outcome and the comprehensive oncologic care. He felt well-informed throughout the treatment process and was grateful for the multidisciplinary approach that enabled successful tumor removal while minimizing risks.

## Data Availability

The original contributions presented in the study are included in the article/Supplementary Material, further inquiries can be directed to the corresponding author.
